# Behavioral Disturbances in Dementia and Beyond: Time for a New Conceptual Frame?

**DOI:** 10.3390/ijms20153647

**Published:** 2019-07-25

**Authors:** Federico Ambrogio, Lucia Anna Martella, Patrizio Odetti, Fiammetta Monacelli

**Affiliations:** 1 Geriatrics Clinic, Department of Internal Medicine and Medical Specialties (DIMI), University of Genoa, 16132 Genoa, Italy; 2 IRCCS Ospedale Policlinico San Martino, 16132 Genoa, Italy

**Keywords:** vascular dementia, Alzheimer’s disease, mixed dementia, behavioral disturbances clusters, antipsychotics

## Abstract

Alzheimer’s disease and vascular dementia are estimated to be the most common causes of dementia, although mixed dementia could represent the most prevalent form of dementia in older adults aged more than 80 years. Behavioral disturbances are common in the natural history of dementia. However, so far, there is a paucity of studies that investigated the causal association between behavioral psychological symptoms of dementia and dementia sub-types, due to the high heterogeneity of methodology, study design and type of clinical assessment. To understand the scant evidence on such a relevant clinical issue, it could be hypothesized that a new shifting paradigm could result in a better identification of the relationship between behavioral disturbances and dementia. This narrative review provides an update of evidence on the behavioral patterns associated with different dementia sub-types and offers a potential future perspective as common ground for the development of new translational studies in the field of behavioral disturbances in dementia and the appropriateness of psychoactive treatments.

## 1. Introduction

Dementia is expected to affect approximately 42.3 million people worldwide by 2020 [1] and Alzheimer’ s disease (AD) is estimated to be the first cause of dementia whereas vascular dementia (VaD) is estimated to be the second most common type, with at least 20% of all cases of dementia [2]. However, with aging, the mixed dementia (MixD) subtype is projected to be the most prevalent type of dementia, especially in older adults aged 80 years and more, ranging from 20–25% to 35% of all cases of dementia. In line with that and from a neuropathological standpoint, Jellinger et al. [3,4] reported that autopsies on *oldest old* patients aged more than 85 years confirmed the high prevalence of mixed brain neurodegenerative conditions, including MixD, synucleinopa-thy, TDP-43 protein accumulation, and astrogliopathy.In a wider conceptual framework, vascular cognitive impairment (VCI) could be considered the first clinical manifestation of VaD, as proposed by Sachdev [5] in 1999, and it includes a series of cognitive deficits of vascular origin. Later, O’Brien et al. [6] broadened this original conceptual framework to all forms of cognitive impairments associated with any cerebrovascular disease and/or vascular brain injury. Wiesmann et al. [7] defined VCI as a cognitive deficit that included at least a single impaired cognitive domain without any impairment of activities of daily living (ADL) [8]. Moreover, VCI could represent the early sign of different clinical conditions, ranging from post-stroke dementia, hypoperfusion dementia, multiple microinfarcts to MixD. Several recent studies pointed out that vascular risk factors are associated with different forms of dementia and that most forms of dementia could be considered a true extension of the inner vascular disease [9].

In addition, the presence of behavioral and psychological symptoms (BPSD) may be associated with the early onset of VCI, although with ambiguous evidence. In particular, the pattern of distribution of behavioral disturbances in VCI showed that apathy, irritability/agitation, depression, anxiety and eating disorders, were the most prevalent symptoms [10].

Indeed, growing evidence indicates that BPSD are common in patients with dementia across the natural history of the disease, representing a wide spectrum of non-cognitive symptoms. Their presence is considered a predictor of poorer clinical outcomes [11], including multimorbidity, disability, poorer quality of life, higher caregivers’ burden [12,13], and institutionalization [14]. In the huge spectrum of behavioral disturbances associated with dementia, it is generally accepted that a series of neurotic symptoms (anxiety, depression, irritability, psychomotor agitation), psychotic symptoms (delusions, hallucinations), apathy, disinhibition, aberrant motor behavior and circadian rhythms disturbances (sleep disorders and eating abnormalities) [15] may vary in intensity and clusters according to the natural history of brain neurodegeneration.

In particular, it could be hypothesized that the clustering and patterns of BPSD may vary on the basis of the etiopathogenetic of dementia. As a result, neurobehavioral disturbances may change in patterns and severity according to the deficits of specific neuroanatomic circuits and/or disconnections. Indeed, patients with VaD may early experience depression, that is generally characterized by a higher clinical burden of symptoms, with reduced antidepressant response. In line with these notions, it could also be conceived that specific BPSD patterns could be associated with different dementia sub-types on the basis of a common neuroanatomic background, although the multifactorial origin of behavioral disturbances makes this putative association a real challenge.

## 2. Behavioral Disturbances and Dementia Sub Types: Vascular Dementia (VaD), Alzheimer’s Disease (AD) and Mixed Dementia (MixD)

So far, there has been a paucity of studies that investigated the causal association between BPSD and dementia sub-types, due to the high heterogeneity of study design, with methodology and population biases and the lack of systematic clinical assessments (see Table 1 for summary). Sultzer et al. [16] investigated the patterns of behavioral disturbances in patients with AD, that included a set of patients comorbid for brain vascular disease and compared them to those affected by VaD. The results showed that VaD patients had increased blunted affect, depression, emotional withdrawal, motor retardation, decreased motivation, anxiety and somatic concerns. In addition, VaD was associated with higher severity of behavioral symptoms and lower response rate to usual anti-depressant and/or psychoactive treatments. An inverse correlation between the cognitive status (as measured by MMSE) [17] and behavioral disturbances (as measured by Neurobehavioral Rating Scale) [18] was also reported. Santos et al. [19] showed that patients with VaD experienced apathy, irritability, anxiety, depression and disinhibition that were the most common neuropsychiatric symptoms (NPS), compared to patients with MixD. Moreover, this disrupting behavioral cluster occurred in the early stage of VaD natural history, although no association with the duration of the disease was observed. 

From a pathogenetic view point, an impairment of the neuro-affective circuits, due to the progressive neurodegeneration, could be hypothesized. 

Hargrave et al. [20] compared the type and clusters of behavioral symptoms in AD dementia subtype compared to VaD subtype. A decreased affect/withdrawal and psychomotor retardation were the most prevalent and disrupting behavioral symptoms in patients with VaD. Although the presence of white matter disease (WMD) was considered responsible for the behavioral disruption, the severity of psychomotor retardation in VaD patients was not correlated with the degree of WMD cortical burden [21,22].

It is noteworthy that the study underpinned the need to include the contribution of additional neurobiological factors to better understand the relation between infarct locations and the progression of affective changes in dementia.

Moreover, Saz et al. [23], in a large population-based sample, analyzed the different behavioral patterns of AD patients compared to those with VaD. In particular, AD patients had a higher rate of “negative-like” symptoms such as anhedonia, and psychomotor retardation whereas VaD patients reported increased “affective-like” symptoms. In addition, psychotic symptoms were most common in VaD dementia while AD patients experienced irritability, dysphoria, delusions, anxiety, and misperceptions. 

Sadak et al. [24] compared the prevalence of four BPSD clusters (agitation, aggression, depression, anxiety, irritability and dysphoria) across dementia etiologies, using AD as the reference type of dementia. The main findings indicated that the prevalence of target BPSD varied according to etiology and the severity of dementia and, namely, AD patients had the lowest prevalence of selected BPSD, while overlapping AD and VAD did not display any higher rate of BPSD patterns.

In keeping with that, Caputo et al. [25] reported a decreased rate of anxiety in VaD patients as compared with patients with AD patients, but no difference in terms of depression and apathy was observed between these two types of dementia. Moreover, Ballard et al. [26] indicated that depression, anxiety and⁄or apathy were the most common behavioral disturbances associated with VaD. Furthermore, Anor et al. [27] compared the behavioral patterns associated with AD, VaD and MixD, respectively, indicating that patients with VaD showed increased psychomotor agitation, sleep disturbance and depression. In contrast, D’Onofrio et al. [28] reported increased agitation/aggression and irritability/lability in patients with AD compared to VaD patients. 

Echávarri et al. [29] did not observe any difference in the behavioral presentation according to specific dementia sub-types. 

Groves et al. [30] observed that patients with VaD were more likely to experience long-standing depression with functional impairment on a long-term observational period, although AD patients were also observed to experience some degree of sleep disorders and depression according to the neurodegenerative trajectory.

In line with all the aforementioned notions, it could be hypothesized that patients with VaD have highly disrupted behavioral disturbances compared to AD and/or MixD patients.

Namely, the putative association of specific behavioral clusters to VaD seems to include psychomotor retardation, anxiety/depression with guilt and somatic concern, early insomnia and general somatic complains [31], although the speculative nature of this association limited the generalizability of the findings.

## 3. Neuroanatomic Findings Between Behavioral and Psychological Symptoms (BPSD) and Dementia Sub-Types (VaD, AD and MixD)

The use of neuroimaging allows the identification of specific neuroanatomic circuits that may be potentially involved in the onset of disruptive behaviors associated with different dementia subtypes. The impairment of the neuroanatomic circuits, due to progressive neurodegeneration refers to the pathological changes in the frontal-subcortical pathways and frontal lobe hypoperfusion, along with the dysfunction of dopaminergic, serotonergic, and noradrenergic neurotransmission. Alterations of these systems are reported to be the pathophysiological background for various behavioral disturbances [32,33]. In addition, focal brain injury or white matter disease, periventricular white matter hyperintensities (WMH), small vessel disease and interruption of subcortical circuit have been also considered the pathogenetic vulnus for the onset of behavioral disturbances in VaD or MixD dementia [34].

However, Brown et al. [32] showed that the older age itself was an indirect risk factor for the presence of WMH signals as a result of vascular changes associated with comorbid medical clinical conditions.

Moreover, Anor et al. [27] observed that VaD patients had a lower rate of WMH frontal cortical burden compared to patients with AD or MixD but this reduced WMH burden was associated with higher disrupted behaviors, including irritability. In contrast, the presence of higher frontal WMH burden was associated with decreased behavioral disturbances, indicating the loss of synaptic connections in the frontal lobe, as a result of WMH insults, the neuroanatomic leakage responsible for the aforementioned symptomatology association. 

Moreover, a significant trend between increased behavioral disturbances and higher WMH right frontal deposition was observed. In particular, AD patients with higher right frontal lobe WM deposition experienced an increased rate of psychotic symptoms such as delusions, compared to those patients with MixD. However, VaD patients with reduced left lobe WMH burden experienced a decreased rate of neurotic symptoms such as irritability.

Ting et al. [35]. observed that the presence of cortical microinfarcts and small vessels disease was associated with the onset of psytha chosis in AD patients, although the magnitude of this association has not been clearly defined, due to the multifactorial origin of behavioral disturbances and the heterogeneous clinical phenotype of old age patients with dementia. Indeed, older adults generally had comorbidity and polypharmacy, that may represent important potential confounders for the appropriate diagnosis and clinical management of BPSD.

In keeping with that, WMH was also reported to be a contributing factor for psychosis and it was suggested that the integrity of small vessels of both cortical and subcortical layers was a key determinant factor for the development of psychosis. Similarly, focal frontal brain injury was observed to be associated with behavioral disturbances, including disinhibition and apathy [36]. In particular, apathy was associated with anterior cingulate atrophy [37] while periventricular WMH burden was associated with hallucination, depression and anxiety in both VaD and AD [38].

Recently, a new theoretical framework included white matter disruption by virtue of altered connectivity of WM network, especially in the frontal and parietal lobes as key determinants of both cognitive and behavioral disturbances in VaD [39].

**Figure 1:**
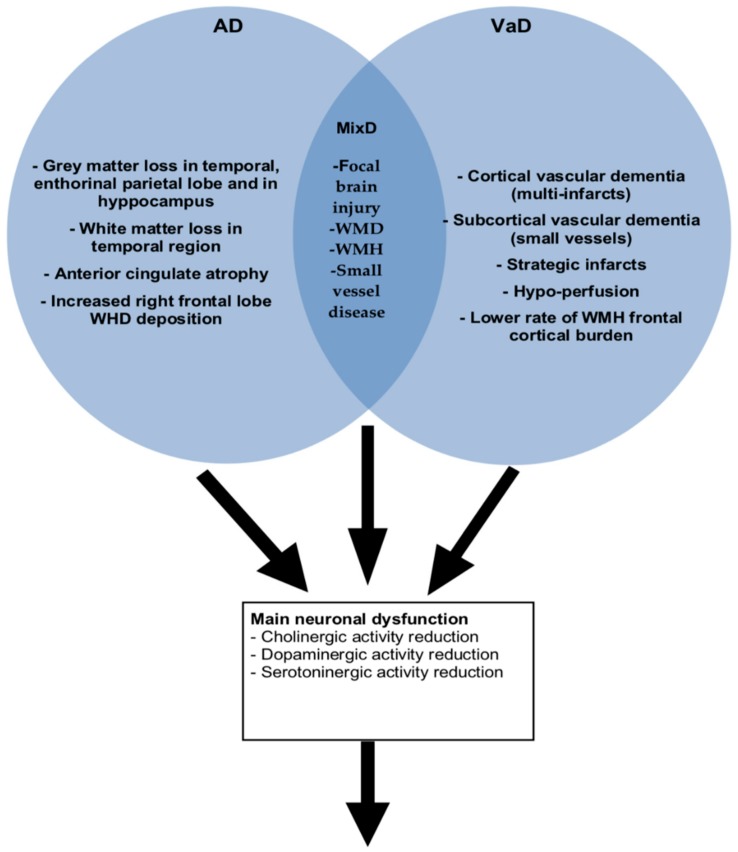
BPSD overlap and patterns according to specific dementia sub-types on the basis of the altered neuroanatomic circuits.

The main neuroanatomic circuits involved in the onset of BPSD are illustrated in Figure 1.

It is noteworthy that a correlation between polymorphisms of apolipoprotein E (APOE) and low-density lipoprotein receptor-related protein gene (LRP) and BPSD patterns in AD and VD dementia has been recently observed. Namely APO epsilon 4 frequency of both AD and VD patients experiencing BPSD was higher than controls. This finding underpinned some similarity between the pathogenesis of AD and VD sub-types, including the pathogenesis of BPSD patterns where psychotics symptoms showed a substantial correlation with APO E epsilon 4 in both AD and MixD sub-types. Indeed, this genetic carriage may promote the deposition of fibrillar amyloid β protein, affecting the amyloid β clearance, with the increase formation of neurofibrillary tangles. In contrast no difference was observed between LPR carriage in AD and VD sub-types [40].

## 4. Antipsychotic Use across Dementia Sub-Types (VaD, AD Versus MixD)

The use of antipsychotics is common in the natural history of dementia to treat behavioral disturbances, although their use may cause additional morbidity and disability in patients with pre-existing clinical vulnerability. It is well known that antipsychotics are associated with worsening cognitive performance, disability, increased caregiver burden, unfavorable clinical outcomes, including falls, anticholinergic burden and delirium [41,42,43,44], hypotension, sedation, and extrapyramidal symptoms. The 2016 American Psychiatric Association (APA) Practice Guidelines provided recommendations for the treatment of agitation or psychosis in patients with dementia, suggesting a comprehensive, person-centered, non-pharmacological approach [45]. 

In line with that, non-pharmacological interventions for behavioral disturbances should be advocated as the first line of therapeutic interventions. In particular, a recent metanalysis showed the clinical benefit of different non-pharmacological approaches on functional status in patients with moderate to advanced dementia. Namely, these interventions included exercise therapy, light therapy, music therapy, massage therapy and multi component therapy [46]. Similarly, Abraha et al. [47]. provided an extensive overview on non-pharmacological interventions to treat BPSD in older patients with dementia, indicating that music therapy and behavioral management techniques were effective in reducing BPSD. However, the heterogeneous definition and application of the same type of intervention affected the generalization of the findings. 

In line with that, Wang et al. [48] categorized three main types of non-pharmacological interventions including, respectively, sensory, cognition and movement-oriented approaches to assess the quality of evidence in treating BPSD in patients with dementia. Indeed sensory-orientated interventions reported the most consistent clinical benefits; however, the ability of the person with dementia to fulfill the requests and to participate successfully in the approach underpinned how tailored interventions are needed for these patients, on the basis of their individualized cognitive abilities. 

Moreover, Caspar et al. [49] developed a unifying heuristic model to improve the understanding of effective non-pharmacological treatment modalities for BPSD. The identification of the caring environment, the development of care skills and maintenance, and the individualization of care represented the key relevant interventions associated with reduced BPSD as well as with increased clinical benefit for those highly vulnerable patients.

Antipsychotics are recommended within this approach as appropriate for the treatment of agitation and other NPS in patients with dementia, when symptoms are severe, threatening, and/or cause significant patient distress. However, at present, no medication has been approved by the United States (US) Food and Drug Administration (FDA) for the treatment of NPS associated with AD. In addition, the US Food and Drug Administration issued a warning on the increased incidence of adverse cerebrovascular events in patients treated with antipsychotics as an off-label treatment for behavioral disturbances in dementia [50]. 

So far, some body of evidence indicates that atypical antipsychotics displayed a better safety and tolerability profile than typical antipsychotics [51,52,53]. Wooltorton et al. [54] and Mowat et al. [55], respectively, claimed that, although the inappropriate use of antipsychotic medications is a critical issue in persons with dementia, the identification of the clinical benefit to risk ratio for the use of antipsychotics in dementia is highly recommended to tailor the therapeutic interventions on an individualized basis. Up to date, there is scant evidence on the antipsychotic prescribing treatment patterns and burden of behavioral disturbances in patients with different dementia sub-types and this is especially true in the prescribing patterns for VaD and MixD. Namely, Moretti et al. [56], assessed the role of olanzapine in patients with subcortical VCI and showed its effective and safety profile on anxiety, with neglected side effects including sedation, postural instability, and postural hypotension. Moreover, no serious anticholinergic effect was observed.

Alfonso et al. [57] showed that risperidone was an effective treatment for both VaD and MixD sub-types and it is, up to date, the sole direct comparison between specific antipsychotic prescribing patterns in different dementia sub-types. The use of risperidone at a mean dose of 1.6 mg/day for 6 months was of clinical benefit in the treatment of aggression, psychomotor agitation and psychosis. A reduction of apathy/indifference and depression/dysphoria was also observed. 

Risperidone was considered to be a well-tolerated drug with respect to the cardiovascular profile and extrapyramidal symptoms, with hypotension and sedation as mild reported side effects. Table 2 illustrates the few evidence on different antipsychotic prescribing patterns on the basis of specific dementia sub-types. Notwithstanding that, the evidence is scant and fragmentary and does not allow any categorization of behavioral disrupted clusters in the presence of specific forms of dementia.

Suh et al. [58] compared haloperidol with risperidone in patients with AD, VD and MixD, indicating that both antipsychotics were of clinical benefit in reducing BPSD. However, risperidone better improved patient’s clinical global impression (Clinical Global Impression of Change scale CGI-C), with a safer profile in terms of reduced risk of antipsychotic-induced parkinsonism.

Recently, Davies S et al. [59] developed an overarching guide to sequential use of psychoactive drugs, including antipsychotics, in order to treat agitation and aggression in AD, Mix D and VD. This evidence-based algorithm was weighed on the basis of safety, tolerability, efficacy, easy to use, time to onset of clinical effect and strength of evidence. In addition, titration schedules, adjustment for patient’s frailty and baseline assessment and/or discontinuation of potentially exacerbating drugs were also considered. It is noteworthy that risperidone, quetiapine and aripiprazole were the most recommended antipsychotics in the over-mentioned types of dementia. Namely, risperidone had the strongest evidence for the treatment of BPSD symptoms in AD and MixD [60,61,62,63], although it carries higher cardiovascular and metabolic risks.

Quetiapine has a considerable weaker evidence for the treatment of BPSD in AD and MixD types, compared to risperidone. However, a previous metanalysis [64] showed the clinical benefit of quetiapine compared to placebo on overall clinical patient’s improvement and on generalized anxiety disorder. This clinical profile makes quetiapine alongside aripiprazole as a first line option for the proposed treatment algorithm.

Moreover, aripiprazole was included as an alternative to quetiapine for the treatment of psychosis in patients AD and MixD [65].

It is noteworthy that haloperidol was excluded from the first line of recommended antipsychotics in the proposed algorithm due to safety concerns, as recently reported in a retrospective study of nursing home resident with AD and MixD sub-types [66,67], where conventionally antipsychotics significantly increased the risk of both mortality and hip fracture. In line with that, the authors speculated that the treatment of patients with AD and/or MixD, experiencing agitation and aggression with the aforementioned antipsychotics may be of some clinical benefit. However, the appraisal of this evidence does not involve systematic reviews but it is based on reviewer’s synthesis of drug’s acceptability, applicability and easy to use.

## 5. Discussion and Conclusions

So far, few studies have investigated the behavioral disrupted patterns associated with different dementia sub-types along with the specific antipsychotics prescribing patterns and the present review provides an updated evidence on this challenging and underestimated clinical issue.

In particular, the main findings showed that VaD patients are more likely to experience a specific neuropsychiatric pattern that is characterized by affective behaviors such as depression, psychomotor retardation, anxiety, apathy and affective withdrawal with a lower response rate to usual antidepressants [68]. From a clinical viewpoint, it could be hypothesized that apathy and depression could be considered two overlapping and misleading clinical conditions. In particular, the appropriate diagnosis of apathy has to date been underreported and undertreated, due to several pathophysiological and diagnostic pitfalls. In turn, the inadequate identification of apathy, especially in VaD patients could, at least partially explain the inherent ineffectiveness of antidepressants therapy in those patients [69]. 

It is generally accepted that several neurodegenerative diseases may be associated with different behavioral disturbances; however, so far, few studies have examined differences in BPSD between dementia sub-types and the results have been quite equivocal [70,71,72,73,74,75,76,77].

However, the available evidence is too limited to gather a comprehensive evaluation of the neuroanatomic pathways that may define the onset of clustered BPSD in specific dementia sub-types. Moreover, this same limitation makes better understand how the identification of appropriate prescribing antipsychotics treatments for selected behaviors underlying a specific sub-type of dementia has been unfeasible up to now.

In line that, a number of issues in assessing the wide spectrum of BPSD in dementia may count for the paucity in results among studies, making the evidence even more controversial.

It is noteworthy that the spectrum of behavioral disturbances in dementia tends to fluctuate over time and each BPSD domain, in terms of pattern, clusters, persistence and intensity over time may be highly individualized on the basis of patients’ clinical phenotype and of the multifactorial origin of disrupted behaviors themselves.

Similarly, this huge heterogeneity in the clinical presentation of behavioral disturbances may be the result of different underlying neurobiological correlates, including the specific form of brain neurodegeneration and its related stage. Additionally, the presence of comorbidity, the individual burden and severity of vascular cerebral load, the failure of synaptic connections and neurotransmission and the ageing process itself may count for additional multicomponent factors, adding further degrees of clinical complexity to the natural history and clinical trajectory of BPSD in different dementia sub-types.

Moreover, although it has been estimated that a higher percentage of patients with dementia experience BPSD over the course of their illnesses, the low epidemiological rate of BPSD in dementia claims for a significant underreporting and a underdiagnosis [68]. This turns out to be especially true for the identification of different pathways to BPSD in different dementia sub-types. 

Furthermore, on the one hand, the rigorous and longitudinal assessment of behavioral profiles in dementia could enable the development of more effective treatments, assessing the appropriateness and tailored use of antipsychotics as well. On the other hand, the current failure in the identification of different patterns for behavioral clusters, according to different forms of dementia, may unmask a major conceptual pitfall. In line with this assumption, this narrative review seems to indirectly confirm that the co-occurrence of multiple neuropathological disorders is common in persons with dementia, especially with advancing age, and this paradigm shift could be applied to the understanding of behavioral disturbances associated with dementia as well. 

So far, the underlying neuropathology of dementia has been poorly established and it could differ significantly from the pathogenesis implied by the clinical diagnosis. Thus, it could be hypothesized that the mere categorization of specific dementia sub-types may serve as a useless platform for the development of studies that are expected to unravel the pathogenesis of dementia, its natural course, including the spectrum of behavioral disturbances. 

An alternative approach is to focus on common molecular mechanisms rather than single specific disease processes that can lead to brain neurodegeneration. In keeping with that, Mallucci et al. [78] developed the unfolded protein theory according to which a common misfolded protein defect primarily occurred in the endoplasmatic reticulum of neurons cells, spreading in between and through cells and ultimately fostering the process of brain neurodegeneration. This theory could be considered an umbrella for different neurodegenerative disorders, including Alzheimer’s disease, various tauopathies, prion disease, idiopathic Parkinson’s disease and the sporadic amyotrophic lateral sclerosis. Whatever the exact mechanism is, the resultant cell-to-cell spreading may favor the metabolic derangement of neurons and astroglia’s cells, boosting brain cellular shutdown. This same detrimental mechanism may be at the basis of the major driving force behind both brain neurocognitive degeneration and behavioral disturbances, due to the sharing of neuroanatomic circuits deficits and neuronal/neurotransmitters disconnections [79].

In line with this notion, it could be hypothesized, although highly speculative in nature, that the aforementioned common molecular background for brain neurodegeneration could be also common ground for behavioral disturbances associated with dementias, in terms of pattern clusters and intensity, irrespective of the categorization in single dementia sub-types. Similarly, a fine and unexplored “switching on/off” mechanism may modulate the onset and progression of both dementia and behavioral disturbances, drawing substantial different patterns across the natural history of brain neurodegenerations.

Thus, in an era of crisis for the treatment of dementia and in presence of scant evidence on the appropriateness of psychoactive treatments for such a huge spectrum of behavioral disturbances, the pursuit of a shifting paradigm for BPSD in dementia could offer a new field of research that has real translational value to provide better quality of care in the near future.

## Figures and Tables

**Table 1:** Summary of the evidence on the different neuropsychiatric symptoms associated with vascular dementia (VaD), Alzheimer’ s disease (AD) and mixed dementia (MixD) sub types.

Authors (Year)	Study Design	Study Population	*N*	Results	Reference
Sultzer et al. (1993)	Observational VaD versus AD	American older adults	104	Patients with VaD had more severe behavioural retardation, depression and anxiety.	[16]
Hargrave et al. (2000)	Observational on patient with VaD versus AD.	American older adults	378	Decreased affect/withdrawal and psychomotor retardation were the most prevalent symptoms in patients with VaD.	[20]
Saz et al. (2009)	Cross sectional VaD versus AD	Spanish (age >55 yrs)	4.803	AD patients had a higher rate of “negative-type” (anhedonia, psychomotor retardation). VaD patients reported increased “affective-type” symptoms.	[23]
Ballard et al. (2000)	Prospective VaD versus AD	English older adults	184	Anxiety and depression were more common in patients with VaD.	[26]
Echávarri et al. (2013)	Retrospective VaD versus AD	Spanish older adults	80	Agitation, depression and anxiety in both groups no significance differences.	[29]
Groves et al. (2000)	Retrospective VaD, versus AD	American (mean age 76 yrs)	517	VaD patients are more depressed and functionally impaired.	[30]
Santos et al. (2018)	Retrospective VaD versus MixD	Brazilian older adults (>60 yrs)	53	Patients with VaD had more apathy, irritability, anxiety and depression. Association between NPS and mild to moderate dementia.	[19]
Anor et al. (2017)	Observational VaD versus AD versus MixD	Canadian older adults	180	VaD patients had higher agitation sleep disturbances, depression and aberrant motor behaviors.	[27]
Caputo et al. (2008)	Observational VaD versus AD and Lewy Body dementia	Italian older adults	921	VaD had less disrupted behaviors.	[25]
D’Onofrio et al. (2012)	Observational	Italian older adults	302	AD patients show increased agitation/aggression and irritability/lability.	[28]
Sadak et al. (2014)	Retrospective VaD, Lewy body dementia, Frontotemporal dementia versus AD	Americans (>65 yrs)	3768	Prevalence of targeted behavioral and psychological symptoms (BPSD) varied according to the aetiology and severity of dementia.	[24]

**Table 2:** Antipsychotic prescription patterns according to specific dementia sub-types (VaD, AD and MixD).

Type of Antipsychotics	Type of Dementia	Mean Dose	Observation Frame	Targeted Symptoms	Side Effects	References
Olanzapine	Vascular cognitive impairment (VCI)	2.5–5 mg/day	6 months	Anxiety	somnolence, postural instability, and postural hypotension.	Moretti et al. [56]
Risperidone	VaD, AD, Mixed	1–2 mg/day	6 months	Aggression, agitation, apathy, depression dysphoria.	Hypotension sedation, paraesthesia.	Alfonso et al. [57]
Risperidone	VaD, AD, Mixed	0.95 mg/day	12 weeks	Aggression, psychotic symptoms	Somnolence, urinary tract infections.	Brodaty et al. [59]
Aripiprazole	AD, VaD, MixD	2.5–10 mg/day	10 weeks	Agitation, psychotic symptoms	Cerebrovascular adverse events.	Mintzer et al. [65]
Risperidone vs. Haloperidol	VaD, AD, Mixed	1–1.5 mg/day	18 weeks	Aggression, anxiety (Risperidone)	Extrapyramidal symptoms (Haloperidol).	Suh et al. [58]
Quetiapine	AD, VaD, Lewy body dementia	300 mg/day	12 weeks	Psychotic symptoms	NA	Cheung et al. [64]

## Abbreviations

VaD	vascular dementia
MixD	mixed type dementia
VCI	vascular cognitive impairment
ADL	activities of daily living
BPSD	behavioral and psychological symptoms of dementia
NPS	neuropsychiatric symptoms
AD	Alzheimer’s disease
MMSE	mini-mental state examination
WMD	white matter disease
WMH	white matter hyperintensities
BEHAVE-AD-K	Behavioral Pathology in Alzheimer’s Disease Rating Scale, Korean version
